# Isoliensinine induces dephosphorylation of NF-κB p65 subunit at Ser536 via a PP2A-dependent mechanism in hepatocellular carcinoma cells: roles of impairing PP2A/I2PP2A interaction

**DOI:** 10.18632/oncotarget.9603

**Published:** 2016-05-26

**Authors:** Guangwen Shu, Lang Zhang, Shanqing Jiang, Zhuo Cheng, Guan Wang, Xu Huang, Xinzhou Yang

**Affiliations:** ^1^ School of Pharmaceutical Sciences, South-Central University for Nationalities, Wuhan, PR China

**Keywords:** isoliensinine, hepatocellular carcinoma, PP2A, I2PP2A, NF-κ B

## Abstract

Our previous study discovered that isoliensinine (isolie) triggers hepatocellular carcinoma (HCC) cell apoptosis via inducing p65 dephosphorylation at Ser536 and inhibition of NF-κB. Here, we showed that isolie promoted p65/PP2A interaction *in vitro* and *in vivo*. Repression of PP2A activity or knockdown of the expression of PP2A-C (the catalytic subunit of PP2A) abrogated isolie-provoked p65 dephosphorylation. I2PP2A is an endogenous PP2A inhibitor. Isolie directly impaired PP2A/I2PP2A interaction. Knockdown of I2PP2A boosted p65/PP2A association and p65 dephosphorylation. Overexpression of I2PP2A restrained isolie-induced p65 dephosphorylation. Untransformed hepatocytes were insensitive to isolie-induced NF-κB inhibition and cell apoptosis. In these cells, basal levels of I2PP2A and p65 phosphorylation at Ser536 were lower than in HCC cells. These findings collectively indicated that isolie suppresses NF-κB in HCC cells through impairing PP2A/I2PP2A interaction and stimulating PP2A-dependent p65 dephosphorylation at Ser536.

## INTRODUCTION

Hepatocellular carcinoma (HCC) is one of the most common human malignant diseases and the second most common cause of cancer-related mortality worldwide [1]. Patients with late-stage HCC currently have to rely on systematic chemotherapy [2]. However, prognosis of patients taking chemotherapeutic drugs for HCC is severely affected by the toxic side effects of the medication and the drug resistance of HCC cells [3]. It is therefore imperative to search for novel molecule targets and chemical entities that can be used in HCC chemotherapy.

Hyperactivation of nuclear factor κB (NF-κB), a family of multifunctional transcription factors, stimulates the onset and progression of HCC [4, 5]. The heterodimer of NF-κB p65 and p50 subunit is a common form of NF-κB. In normal cells, NF-κB is precisely regulated by highly orchestrated molecular mechanisms. Phosphorylation of p65 is a critical contributor to the regulation of NF-κB. For example, p65 can be phosphorylated at Ser536, enhancing its transcriptional potential [6]. Ser536-phosphorylated p65 can be dephosphorylated by protein phosphatases such as protein phosphatase 2A (PP2A) [7]. PP2A-C is the catalytic subunit of PP2A and an indispensible member of PP2A core enzyme [8]. Abnormal expression of PP2A subunits or deficiency of PP2A activity promote carcinogenesis or predict dismal prognosis of some malignant diseases [9–11]. I2PP2A, also known as SET, is an endogenous inhibitor of PP2A. I2PP2A physically binds to PP2A and inhibits its protein phosphatase activity [12]. I2PP2A is involved in a series of human cancers [13]. In non-small cell lung cancer, high levels of I2PP2A are correlated with poor prognosis [14]. However, roles of I2PP2A in HCC still remain to be fully elucidated.

Many literatures have reported the remarkable properties of phytochemicals and their derivatives as anticancer reagents [15–18]. Isoliensinine (isolie) is an alkaloid derived from embryos of *Nelumbo nucifera*. It has been discovered that isolie induces HCC cell apoptosis by stimulating p65 dephosphorylation at Ser536. Isolie has no detectable toxic effects on untransformed hepatocytes or tumor-bearing animals [19]. The current study is designed to further explore how isolie promotes p65 dephosphorylation in HCC cells. These findings may facilitate understanding of the effects of isolie on HCC cells and untransformed hepatocytes at the molecular level. These factors are crucial to the potential application of isolie in the clinical treatment of HCC.

## RESULTS

### Isolie promoted p65/PP2A interaction and inhibition of PP2A activity suppressed isolie-induced p65 dephosphorylation at Ser536

**Figure 1:**
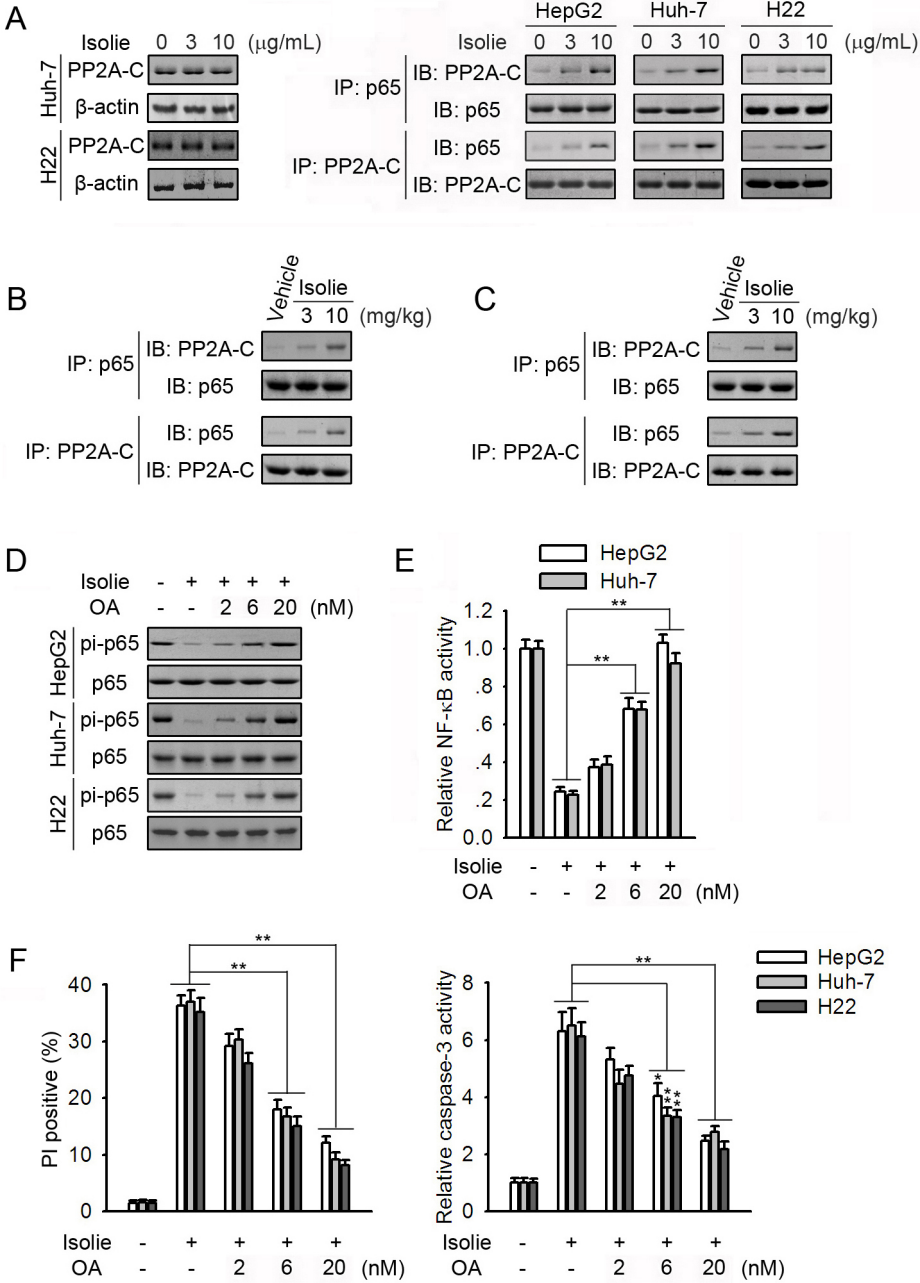
PP2A participated in isolie-induced p65 dephosphorylation **A.** Indicated HCC cells were treated with different concentrations of isolie. After 24 h, protein levels of PP2A-C (left) and p65/PP2A interaction (right) were determined. **B.** Indicated dosages of isolie or vehicle were intraperitoneally injected into tumor-bearing nude mice over the course of 3 weeks. Finally, effects of isolie on p65/PP2A interaction in Huh-7 tumor tissues were determined. **C.** Kunming mice bearing H22 tumors were treated with indicated dosages of isolie by gavage for 10 d. Then, effects of isolie on p65/PP2A interaction in transplanted tumor tissues were detected. **D, E.** HCC cells were treated with 10 μg/mL isolie and indicated concentrations of OA. After 24 h, phosphorylation of p65 at Ser536 was determined (D), and NF-κB activity was measured by NF-κB-dependent luciferase reporter assay (E). **F.** Cells were subjected to the same treatment as described in D and E. Cell apoptosis was quantified by FACS (left) and caspase-3 activity assay (right) 48 h later. * p < 0.05 and ** p < 0.01, compared to the indicated control.

PP2A is a protein phosphatase catalyzing p65 dephosphorylation at Ser536 [20]. Levels of PP2A-C remained unchanged in response to isolie (Figure 1A and 3A, left). However, isolie dose-dependently elevated p65/PP2A binding in HepG2, Huh-7 and H22 HCC cells (Figure 1A, right). In Huh-7 xenograft tumors, isolie promoted p65/PP2A interaction (Figure 1B). In H22 transplanted tumors, isolie stimulated p65/PP2A association (Figure 1C). Okadaic acid (OA) is an inhibitor of PP2A. In HCC cells, OA dose-dependently attenuated isolie-induced dephosphorylation of p65 and inhibition of NF-κB (Figure 1D and 1E). Isolie-induced HCC cell apoptosis was in turn considerably suppressed by OA (Figure 1F).

### Knockdown of PP2A-C reduced p65 dephosphorylation provoked by isolie

**Figure 2:**
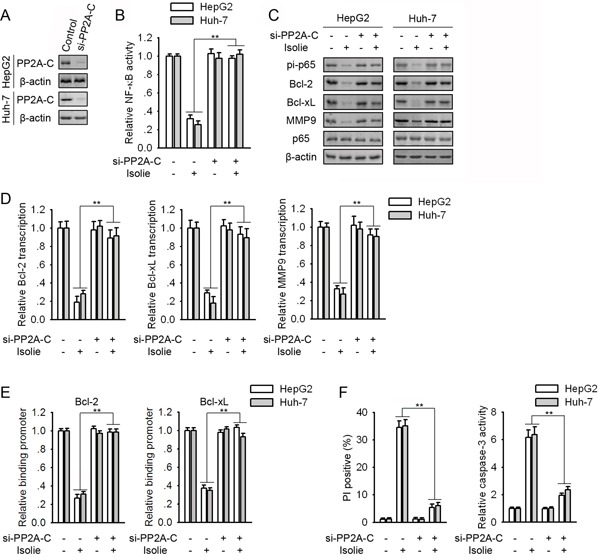
Knockdown of PP2A-C abrogated isolie-induced p65 dephosphorylation and HCC cell apoptosis **A.** HCC cells were transfected with control or PP2A-C specific siRNA. After 48 h, protein levels of PP2A-C were detected. **B, C, D, E.** HepG2 and Huh-7 cells were transfected with control or PP2A-C siRNAs for 48 h. Then, cells were treated with 10 μg/mL isolie. After 24 h, NF-κB activities were detected by luciferase reporter assay (B). Levels of indicated proteins were examined by immunoblotting (C). mRNA levels of Bcl-2, Bcl-xL and MMP9 were detected by real-time PCR (D). Interactions between p65 and promoter regions of indicated genes were determined by ChIP (E). **F.** Indicated HCC cell transfectants were treated with 10 μg/mL isolie. After 48 h, cell apoptosis were quantified by FACS (left) and caspase-3 activity assay (right). ** p < 0.01, versus cells transfected with control siRNA.

To confirm the role of PP2A in isolie-induced NF-κB repression, PP2A-C was knockdown by its specific siRNA (Figure 2A). Knockdown of PP2A-C abolished isolie-induced NF-κB inhibition in HCC cells (Figure 2B). Bcl-2, Bcl-xL and MMP9 are typical NF-κB-responsive genes. In HCC cells transfected with control siRNA, isolie decreased p65 phosphorylation at Ser536 and binding between p65 and promoter regions of Bcl-2 and Bcl-xL encoding genes. Knockdown of PP2A-C abrogated these effects (Figure 2C and 2E). Consistently, in response to isolie, levels of NF-κB-responsive genes were declined in control HCC cells, but remained almost unchanged in PP2A-C knockdown cells (Figure 2C and 2D). Isolie-induced HCC cell apoptosis was also restrained by knockdown of PP2A-C (Figure 2F).

### Isolie impaired the interaction between PP2A and I2PP2A

**Figure 3:**
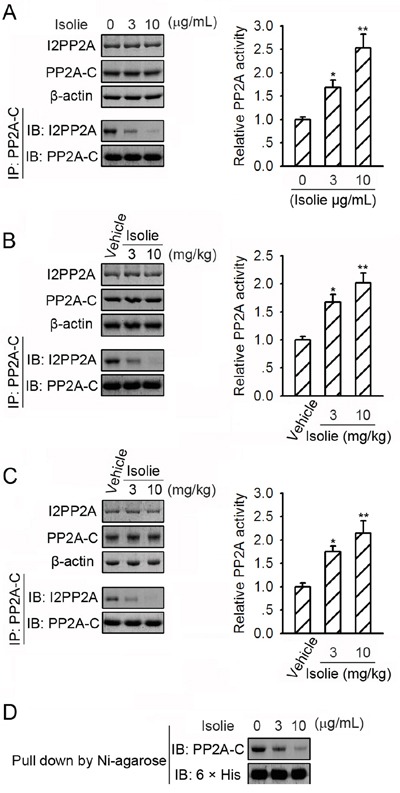
Isolie suppressed PP2A/I2PP2A interaction **A.** HepG2 cells were incubated with indicated concentrations of isolie for 24 h. Then, PP2A/I2PP2A interaction was detected by immunoprecipitation (left). Phosphatase activity of immunoprecipitated PP2A was quantified by phosphatase activity assay (right). **B.** Indicated dosages of isolie or vehicle were injected intraperitoneally into nude mice bearing Huh-7 xenograft tumors. In the end, tumor lysates were prepared and subjected to the same experiments as described in A. **C.** Kunming mice bearing H22 transplanted tumors were treated with indicated dosages of isolie by gavage. In the end, tumor lysates were prepared and subjected to the same experiments as described in A. **D.** Purified 6 × his-tagged I2PP2A proteins already associated with Ni-NTA agarose were incubated with purified PP2A-C proteins and indicated concentrations of isolie. Amounts of PP2A-C proteins bound to I2PP2A proteins *in vitro* were determined by immunoblotting. * p < 0.05 and ** p < 0.01, compared to cells without isolie treatment (A) or tumors dissected from mice treated with saline (B and C).

I2PP2A is an endogenous inhibitor of PP2A. I2PP2A was found to bind to PP2A in HepG2 cells. In response to isolie, levels of I2PP2A remained unchanged, but PP2A/I2PP2A interaction was reduced (Figure 3A, left). Phosphatase activity of PP2A immunoprecipitated from HepG2 cell lysates was elevated by the stimulation of isolie in a dose-dependent manner (Figure 3A, right). Isolie also downregulated PP2A/I2PP2A association and increased PP2A phosphatase activity in Huh-7 xenograft tumors and H22 transplanted tumors (Figure 3B and 3C). Purified recombinant 6 × his-tagged I2PP2A protein bound to purified PP2A-C *in vitro*. This interaction was dose-dependently attenuated by isolie (Figure 3D). These data indicated the capability of isolie to directly impair PP2A/I2PP2A interaction.

### Isolie-induced p65 dephosphorylation at Ser536 was triggered by reduced PP2A/I2PP2A interaction

**Figure 4:**
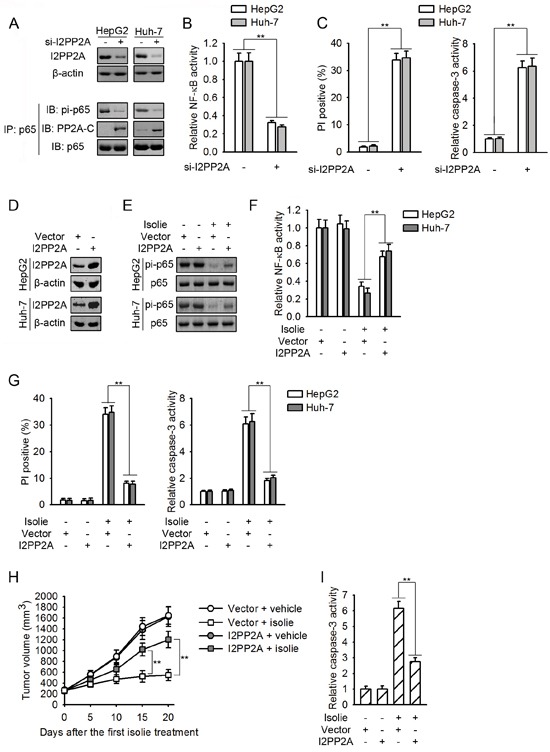
Isolie-provoked p65 dephosphorylation was initiated by decreased PP2A/I2PP2A interaction **A, B, C.** HCC cells were transfected with control or I2PP2A specific siRNA. After 48 h, levels of I2PP2A and effect of I2PP2A knockdown on p65/PP2A association as well as p65 phosphorylation at Ser536 were determined (A). NF-κB activities were measured by luciferase reporter assay (B). After 72 h, cell apoptosis were quantified by FACS (C, left) and caspase-3 activity assay (C, right). **D.** The encoding gene of human I2PP2A was stably transfected into indicated HCC cells, using empty vectors as the negative control. Then, I2PP2A proteins were detected. **E, F.** HCC cell transfectants as indicated were treated with 10 μg/mL isolie. After 24 h, levels of p65 phosphorylation at Ser536 (E) and NF-κB activity (F) were detected. **G.** Indicated HCC cell transfectants were incubated with 10 μg/mL isolie. After 48 h, cell apoptosis was detected by FACS (left) and caspase-3 activity assay (right). **H, I.** Indicated Huh-7 HCC cell transfectants were subcutaneously transplanted into nude mice. When volumes of xenograft tumors reached about 200 mm^3^, mice were treated with 10 mg/kg isolie or vehicle once daily for 20 d by gavage. Tumor growth was monitored every 5 d (H). Finally, xenograft tumor lysates were prepared and subjected to caspase-3 activity assay (I). ** p < 0.01 versus the indicated control.

If isolie-induced p65 dephosphorylation was initiated by the impairment of PP2A/I2PP2A interaction, knockdown of I2PP2A which released PP2A from PP2A/I2PP2A complex should promote p65/PP2A binding and p65 dephosphorylation. As expected, knockdown of I2PP2A stimulated p65/PP2A interaction and decreased p65 phosphorylation at Ser536 (Figure 4A). Constitutive NF-κB activity was in turn inhibited, resulting in HCC cell apoptosis (Figure 4B and 4C). Consistently, overexpression of I2PP2A inhibited isolie-induced dephosphorylation of p65 and suppression of NF-κB (Figure 4D, 4E and 4F). HCC cells that overexpressed I2PP2A were resistant to isolie-provoked apoptosis *in vitro* and in nude mice (Figure 4G, 4H and 4I).

### Expression levels of I2PP2A and cellular responses to isolie

**Figure 5:**
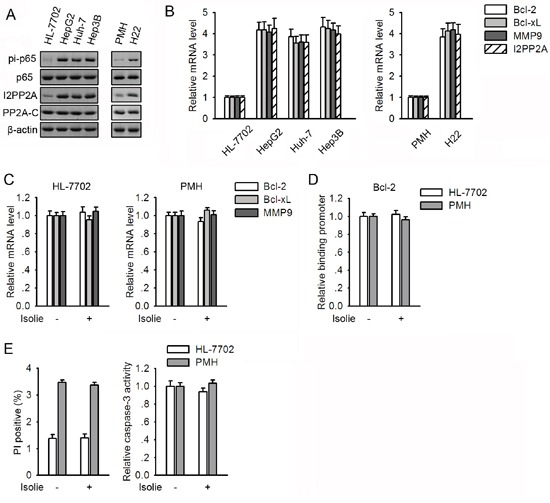
Untransformed hepatocytes showed lower I2PP2A and constitutive NF-κB activity than those in HCC cells and were resistant to isolie **A.** Basal levels of I2PP2A, PP2A-C and p65 phosphorylation at Ser536 in malignant and untransformed hepatocytes were compared. **B.** mRNA levels of NF-κB-responsive genes and I2PP2A in malignant and untransformed hepatocytes were compared. **C, D.** HL-7702 cells and PMHs were incubated with vehicle or 10 μg/mL isolie. After 24 h, transcription of NF-κB target genes and the binding between p65 and Bcl-2 promoter were detected by real-time PCR (C) and ChIP (D), respectively. **E.** PMH and HL-7702 cells were treated with vehicle or 10 μg/mL isolie. After 48 h, cell apoptosis was assessed using FACS (left) and caspase-3 activity assay (right).

Human HL-7702 hepatocytes and mouse primary hepatocytes (PMHs) are considered untransformed hepatocytes. Protein levels of PP2A-C were comparable among untransformed and malignant hepatocytes (Figure 5A). In contrast, levels of I2PP2A in HCC cell lines were substantially higher than in untransformed hepatocytes. Among all these cells, levels of I2PP2A were positively correlated with both p65 phosphorylation at Ser536 and transcription of NF-κB-responsive genes (Figure 5A and 5B).

In HL-7702 cells and PMHs, transcription of NF-κB target genes and the association between p65 and Bcl-2 promoter remained unchanged in response to isolie (Figure 5C and 5D). Moreover, cytotoxic effects of isolie on HL-7702 cells and PMHs were undetectable (Figure 5E). In view of the fact that isolie is a strong inducer of apoptosis in HCC cells in which levels of I2PP2A are higher, these findings indicated a relationship between basal levels of I2PP2A and cellular responses to isolie.

## DISCUSSION

Phosphorylation dynamics of proteins, including NF-κB p65 subunit, are orchestrated by opposing activities of kinases and phosphatases. PP2A-dependent p65 dephosphorylation inhibits NF-κB [21]. In the current study, isolie induced p65 dephosphorylation at Ser536 via the activation of PP2A. In vascular smooth muscle cells, other structurally unrelated compounds provoke p65 dephosphorylation at Ser536 through a similar mechanism [22, 23]. In addition to directly dephosphorylating p65, PP2A is also constitutively recruited to and dephosphorylates IKK in human carcinoma cells [24]. IKK is capable of phosphorylating p65 at Ser536 [25]. It is thus possible that isolie may elevate PP2A-dependent dephosphorylation of IKK, leading to indirect p65 dephosphorylation.

**Figure 6:**
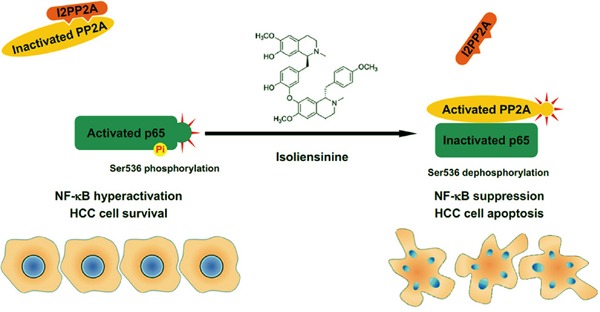
Summary by a schematic panel In untreated HCC cells, I2PP2A binds to PP2A. PP2A/I2PP2A association reduces the phosphatase activity of PP2A and prevents PP2A from binding to NF-κB p65 subunit. These events benefit p65 hyperphosphorylation at Ser536 and hyperactivation of NF-κB, which is important for the survival of HCC cells. Isolie disrupts PP2A/I2PP2A interaction, increases p65/PP2A association and upregulates the phosphatase activity of PP2A. In this way, p65 is dephosphorylated at Ser536, giving rise to the inhibition of NF-κB and subsequent apoptosis of HCC cells.

Elevated protein levels of I2PP2A have been reported in an array of human cancers [26–28]. Similarly, in both murine and human HCC cells, levels of I2PP2A were found to be higher than in their untransformed counterparts. I2PP2A interacts directly with ceramide. This event reduces PP2A/I2PP2A association and elevates PP2A-dependent protein dephosphorylation [29]. *In silico* structural simulation has shown that the direct binding of I2PP2A to ceramide reactivates PP2A and leads to cell death [30]. Here, our experimental data indicated that isolie directly attenuated PP2A/I2PP2A interaction without decreasing levels of I2PP2A. Declined binding between PP2A and I2PP2A increased both p65/PP2A association and the phosphatase activity of PP2A. Overexpression of I2PP2A restrained isolie-triggered dephosphorylation of p65 and apoptosis of HCC cells. These findings indicated that I2PP2A can be a direct target of isolie. In addition to isolie, other structurally unrelated chemicals also enhance the activity of PP2A via interfering with the binding between PP2A and I2PP2A [31, 32]. The molecular pathways underlying isolie-provoked p65 dephosphorylation at Ser536 and subsequent HCC cell apoptosis are schematically summarized in Figure 6.

In HCC cells, knockdown of I2PP2A led to a decrease of NF-κB, and overexpression of I2PP2A inhibited isolie-induced NF-κB inhibition. These observations indicated that I2PP2A can be a functional positive regulator of NF-κB in HCC cells. A series of proteins capable of physically binding to p65 and modulating NF-κB activity has been identified [33–35]. Unlike these factors, I2PP2A promotes NF-κB activity by reducing p65/PP2A interaction. These data provide new insights into the molecular mechanism concerning the regulation of NF-κB. The positive correlation between I2PP2A and NF-κB explains a clinical report showing that upregulated levels of I2PP2A predicts poor prognosis in human leukemia [36].

In malignant and untransformed hepatocytes, protein levels of NF-κB subunit p65 were similar. However, basal levels of p65 phosphorylation at Ser536 and transcription of typical NF-κB-responsive genes were much higher in HCC cells than those in their untransformed counterparts. These data indicated that basal levels of NF-κB activity are higher in HCC cells. Isolie triggered HCC cell apoptosis via suppressing NF-κB. Similarly, an array of previous studies reported that HCC cell apoptosis is associated with reduced NF-κB activity [37–39]. These observations indicate that hyperactivated NF-κB promotes HCC cell survival. Aberrant p65 phosphorylation at Ser536 is also observed in other types of human malignant tissues, not only in HCC [40–42]. Increased basal levels of p65 phosphorylation at Ser536 can thus be a mechanistical contributor to the cancer-related hyperactivation of NF-κB.

Expression levels of I2PP2A were greater in HCC cells than those in their untransformed counterparts. Knockdown of I2PP2A led to HCC cell apoptosis, indicating the crucial role of I2PP2A in the maintenance of HCC cell survival. In addition, higher protein levels of I2PP2A were correlated with higher p65 phosphorylation at Ser536 and higher constitutive NF-κB activity in HCC cells. Lower I2PP2A levels were correlated with lower p65 phosphorylation levels and less basal NF-κB activity in untransformed hepatocytes. These phenomena can be explained by the finding that I2PP2A is a positive regulator of p65 phosphorylation via attenuating p65/PP2A interaction. Moreover, it is reasonable to speculate that, in HCC cells, upregulated expression of I2PP2A is an important contributor to the high basal level of p65 phosphorylation at Ser536 and NF-κB activity. In HCC cells in which levels of I2PP2A were higher, isolie attenuated PP2A/I2PP2A interaction, promoted p65/PP2A association and decreased p65 phosphorylation, leading to NF-κB inhibition and apoptosis. In untransformed hepatocytes with relatively lower levels of I2PP2A, isolie exhibited few effects on NF-κB activity, and these cells were insensitive to isolie-induced apoptosis. Based on these findings, it is feasible to propose hypothesize that isolie selectively exerts its cytotoxic effects on cells that require high levels of I2PP2A to maintain their survival. Suppression of I2PP2A should be evaluated as a strategy for the treatment of HCC.

## MATERIALS AND METHODS

### Reagents and antibodies

Isolie was prepared by methods as described previously [19]. Dulbecco's Modified Eagle Medium (DMEM), fetal calf serum (FCS), penicillin and streptomycin for cell culture were purchased from Invitrogen (Carlsbad, CA, USA). FuGENE reagents for cell transfection were obtained from Roche (Penzberg, Germany). Control siRNA and siRNA specific to human PP2A-C and I2PP2A encoding genes were bought from Santa Cruz Biotechnology (Santa Cruz, CA, USA). Propidium iodide (PI), Trizol reagent and protein G sepharose were bought from Beyotime Biotechnology (Nantong, Jiangsu, PR China). NF-κB-dependent luciferase reporters and pRL-TK plasmids expressing *Renilla* luciferase constitutively which were used as an internal control were purchased from Promega (Fitchburg, WI, USA). Ni-NTA agarose was from Amersham Pharmacia (Buckinghamshire, England). Recombinant human PP2A-C protein was bought from Cayman Chemical (Ann Arbor, MI, US). Matrigel was from BD Biosciences (Bedford, MA, USA). Primary antibodies against p65, PP2A-C, Bcl-2, Bcl-xL, MMP9 and I2PP2A were from Santa Cruz Biotechnology (Santa Cruz, CA, USA). Primary antibodies against β-actin and Ser536-phosphorylated p65 were obtained from Cell Signaling Biotechnology (Danvers, MA, USA). Secondary antibodies conjugated with horseradish peroxidase were bought from ProteinTech Group (Wuhan, Hubei, PR China).

### Cell culture and transfection

Human HepG2 and Huh-7 HCC cells, HL-7702 untransformed hepatocytes and murine H22 ascitic hepatoma cells were cultured in DMEM supplied with 10% FCS, 100 U/mL penicillin and streptomycin. Cells were incubated in a humidified atmosphere containing 5% CO_2_ at 37°C. Primary mouse hepatocytes were prepared and cultured as described previously [43]. To knockdown PP2A-C and I2PP2A, their specific siRNA were transfected into HepG2 and Huh-7 cells using FuGENE. HepG2 and Huh-7 cells stably overexpressing I2PP2A were prepared using a lentiviral system as described previously [44].

### Animals, ethics statement and treatment

Male Kunming mice and athymic nude mice (6-week old, weighting 18 to 22 g) were from the Center of Experimental Animals, Institute of Health and Epidemic Prevention (Wuhan, Hubei, PR China). Mice were housed in standard specific-pathogen-free environment with sterilized food and water supplied *ad libitum*. All of the animal experimental protocols followed here had been approved by the Experimental Animals Care and Use Committee of South-Central University for Nationalities (Wuhan, Hubei, PR China).

Huh-7 cells (or indicated Huh-7 transfectants) were suspended in 0.2 mL of serum-free DMEM containing 50% Matrigel and subcutaneously transplanted into dorsal flanks of nude mice (1 × 10^6^ cells/mouse). Tumor-bearing nude mice were randomly divided into groups (6 mice for each group). When volumes of xenograft tumors reached about 200 mm^3^, animals were intraperitoneally injected with indicated dosages of isolie resolved in normal saline (0.9% NaCl) for 3 weeks, once daily. The vehicle, i.e., normal saline, was used as the negative control. Finally, all tumor-bearing mice were euthanized, and their xenograft tumors were dissected out immediately for further experiments.

HCC-suppressing activity of isolie was also tested in Kunming mice. In this model, 1 × 10^6^ of H22 cells were subcutaneously transplanted into dorsal flanks of Kunming mice. Mice were randomly divided into groups. Each group contained 10 mice. Drug treatment began 24 h later by gavage, once daily. Isolie groups received different dosages (3 and 10 mg/kg). The vehicle control group received normal saline. Ten days later, immediately after euthanizing all animals, transplanted tumors were dissected out and subjected to further analysis.

### Immunoblotting (IB) and immunoprecipitation (IP)

Dissected tumor tissues or cultured cells were homogenized in 0.2 mL of ice-cold lysis buffer containing 50 mM Hepes (pH 7.4), 50 mM NaCl, 5 mM EDTA, 1% Triton X-100, 50 mM NaF, 1 mM Na_3_VO_4_, 10 mM Na_4_P_2_O_7_, 10 mg/mL leupeptin, 10 mg/mL aprotinin, and 1 mM PMSF. After removing insoluble fragments by centrifugation, samples were subjected to sodium dodecyl sulfate polyacrylamide gel electropheresis (SDS-PAGE, 30 μg of protein per lane) and transferred onto nitrocellulose membranes. Membranes were blocked in 5% skim milk in Tris-buffered saline containing 0.1% Tween-20 (TBST) for 30 min and incubated with the indicated primary antibody (1: 2000 dilution) overnight at 4°C. Then, membranes were washed with TBST for 30 min at room temperature and incubated with corresponding horseradish peroxidase-conjugated secondary antibody for 2 h. After 45 min of extensively washing with TBST, protein bands were visualized by enhanced chemiluminescence.

To perform immunoprecipitation assay, cell pellets or tumor tissues were lysed in ice-cold RIPA buffer (phosphate-buffered solution supplied with 1% Nonidet P-40, 0.1% sodium dodecyl sulfate, 0.5% sodium deoxycholate, 50 mM NaF, 1 mM Na_3_VO_4_, 1 mM PMSF, 5 μg/mL aprotinin, 5 μg/mL leupeptin, and 10 mM Na_4_P_2_O_7_). Insoluble fractions were removed by centrifugation at 12000 × g for 15 min at 4°C, and supernatants were pre-cleared with protein G sepharose. Immunoprecipitation assay was performed by incubating the pre-cleared sample with protein G sepharose pre-absorbed with 2 μg of the indicated primary antibody at 4°C for 2 h. After washing sepharose beads extensively with RIPA buffer, sepharose beads were boiled in 20 μL of 1 × SDS-PAGE loading buffer. Proteins eluted from sepharose beads were finally analyzed by immunoblotting.

### Chromatin immunoprecipitation (ChIP)

Relative bindings of p65 proteins to promoter regions of Bcl-2 and Bcl-xL encoding genes were detected by ChIP assay, followed by real-time PCR. ChIP assay was performed by using the corresponding kit bought from Upstate Biotechnology (Boston, MA, USA). Primers for quantifying precipitated DNA are listed below (5′ to 3′). Human Bcl-2 promoter: forward: GGG GAG AAC TTC GTA GCA GT; reverse: GAG AGG GGA CGA TGA AGG AG. Human Bcl-xL promoter: forward: CTC ACC CAG TCT TTG TGC AG; reverse: GGA GGT GGC TGG TAT GGA TT. Mouse Bcl-2 promoter: forward: GGC AAA CCC TCC CCC ACC ACC TC; reverse: CCA CCG GAC CGC TTC AGA CCT C.

### Caspase-3 activity assay

Relative activities of caspase-3 in lysates of cultured cells or tumor tissues were measured using the caspase-3 activity assay kit bought from Beyotime Biotechnology according to the manufacturer's instructions (Nantong, Jiangsu, PR China).

### NF-κB luciferase reporter assay

NF-κB luciferase reporters and pRL-TK plasmids were co-transfected into HCC cells in 24-well plates using FuGENE reagents. After transfection for 24 h, different concentrations of isolie were add and incubated for another 24 h. In the end, intracellular luciferase activities were determined using the Dual Reporter assay system from Promega (Fitchburg, WI, USA).

### RNA isolation, reverse transcription and quantitative real-time PCR

RNA isolation, reverse transcription and quantitative real-time PCR were conducted as described previously [45]. Primers for real-time PCR are listed as follows (5′ to 3′). Bcl-2 (human): forward: GGT GGG GTC ATG TGT GTG G; reverse: CGG TTC AGG TAC TCA GTC ATC C. Bcl-xL (human): forward: GAG CTG GTG GTT GAC TTT CTC; reverse: GCA GTT CAA ACT CGT CGC CT. MMP-9 (human): forward: TGT ACC GCT ATG GTT ACA CTC G; reverse: CGG CAA GTC TTC CGA GTA GT. GAPDH (human): forward: GGA GCG AGA TCC CTC CAA AAT; reverse: GCC ATC ACG CCA CAG TTT C.

### Flow cytometry analysis (FACS) of cell apoptosis

After the treatment as indicated in the text, cells were trypsinized, fixed in 70% ethanol at 4°C for over 2 h and resuspended in PBS containing 20 mM EDTA. Intracellular RNA was then degraded by incubating samples with RNaseA (1 mg/mL) at 37°C for more than 2 h. Finally, cells were stained with PI (30 μg/mL) and subjected to FACS (Becton Dickinson FACSCalibur, Franklin Lakes, NJ, USA).

### PP2A activity assay

The protein phosphatase activity of PP2A was quantified by detecting the generation of free phosphate released from threonine phosphopeptides using the malachite green-phosphate complex assay as described by the manufacturer (Upstate Biotechnology, Boston, MA, USA). HCC cell or tumor lysates were prepared in a low-detergent lysis buffer (1% Nonidet P-40, 10 mM HEPES, 150 mM NaCl, 10% glycerol, 1 mM PMSF, 5 mM benzamidine, and 10 g/mL leupeptin). The lysates were then subjected to immunoprecipitation using the primary antibody specific to PP2A-C. Phosphatase activity assay was performed in a PP2A-specific reaction buffer from Millipore (Billerica, MA, USA) containing 750 mM phosphopeptide substrate. After incubation at 30°C for 10 min, malachite dye was added, and levels of free phosphate were represented by the optical density of the reaction system at 650 nm.

### *In vitro* protein interaction analysis

Recombinant human I2PP2A protein with 6 × his tag were expressed in *Escherichia coli* BL21 strain and purified by affinity chromatography using Ni-NTA agarose. For *in vitro* protein interaction analysis, the binding buffer contained 20 mM Tris-HCl (pH 8.0), 1 mM EDTA, 150 mM NaCl, 10% glycerol and 0.1% Nonidet P-40. Commercialized recombinant PP2A-C protein (1 μg) dissolved in 1 mL of binding buffer were incubated with 2 μg of 6 × his-tagged recombinant I2PP2A protein already bound to Ni-NTA agarose and different concentrations of isolie at 4°C for 3 h. Then, samples were washed 5 times with binding buffer. Finally, the Ni-NTA agarose were eluted in 50 μL of 1 × SDS-PAGE loading buffer. Protein levels of 6 × his-tagged I2PP2A and PP2A-C bound to Ni-NTA agarose were detected by immunoblotting.

### Statistical analysis

Data displayed in our current study are representatives or statistics (mean value ± standard deviation) of results from at least three independent experiments or all experimental animals of each group. Statistical significance was determined by one-way analysis of variance and Dunnett's t tests.
